# Association between exposure to endocrine-disrupting chemicals and polycystic ovary syndrome: a systematic review

**DOI:** 10.61622/rbgo/2026rbgo19

**Published:** 2026-05-29

**Authors:** Diemer Muñoz-Verbel, Eutimio Cueto-Almeida, Albeiro Marrugo-Padilla, Johana Márquez-Lázaro

**Affiliations:** 1 Corporación Universitaria Rafael Núñez Faculty of Health Sciences Cartagena Colombia Corporación Universitaria Rafael Núñez, Faculty of Health Sciences, Cartagena, Colombia.; 2 Corporación Universitaria Rafael Núñez Faculty of Health Sciences, TOXSA Group Cartagena Colombia Faculty of Health Sciences, TOXSA Group, Corporación Universitaria Rafael Núñez, Cartagena, Colombia.

**Keywords:** Endocrine disruptors, Polycystic ovary syndrome, Bisphenol A, Triclosan

## Abstract

**Objective:**

To summarize the available evidence on the association between endocrine disruptors and polycystic ovary syndrome (PCOS).

**Methods:**

A search was conducted in the MEDLINE (via PubMed); Science direct, Scopus and LILIACS databases for relevant studies published between 2013 and 2025. The inclusion criteria covered cohort, cross-sectional, and case-control studies published in English or Spanish. Data extraction and quality assessment were performed using the Joanna Briggs Institute (JBI) checklists, and the results were synthesized descriptively. The articles retrieved were reviewed by two evaluators; 49 were chosen for full-text review, 30 were included in the qualitative analysis.

**Results:**

This systematic review found that bisphenol A (BPA) is the most frequently associated endocrine disruptor with PCOS. This compound interferes with steroidogenesis by mimicking estrogen, which may contribute to insulin resistance and androgen dysfunction. Additionally, phthalates were found to be related to elevated androgen levels and ovarian dysfunction, while triclosan and cadmium were associated with hormonal imbalances affecting ovarian reserve and testosterone levels. Furthermore, perfluoroalkyl and polyfluoroalkyl substances (PFAS) were linked to reduced ovarian reserve and a higher prevalence of PCOS.

**Conclusions:**

The results of this review highlight the role of endocrine disruptors in the pathophysiology of polycystic ovary syndrome, emphasizing the need for further research to identify emerging disruptors and their potential mechanisms in the development of this disease. Therefore, it is crucial to adopt public health measures aimed at minimizing exposure to these compounds, particularly in women who are more susceptible to developing PCOS.

## Introduction

Polycystic Ovary Syndrome (PCOS) is the most common endocrine disorder in women of reproductive age, characterized by chronic oligoovulation or anovulation, hyperandrogenism, and polycystic ovarian morphology.^(1)^ The nature of the disease is heterogeneous, often accompanied by acne, alopecia, and hirsutism.^(2)^ Complications associated with PCOS include obesity, dyslipidemia, insulin resistance, and an increased risk of type 2 diabetes mellitus, cardiovascular disease, endometrial carcinoma, and psychological disorders such as stress and depression.^(2,3)^

Currently, the diagnosis of PCOS remains a topic of controversy in clinical endocrinology due to its wide range of clinical and biochemical presentations, which often complicates accurate diagnosis.^(4)^ The Rotterdam criteria are typically used for identification, proposing the fulfillment of two of the following conditions: 1) Presence of oligomenorrhea or anovulation; 2) Presence of clinical or biochemical hyperandrogenism; and 3) Presence of polycystic ovaries (≥12 follicles in each ovary measuring 2–9 mm) observed by transvaginal ultrasound.^(4,5)^ Additionally, a subclassification of PCOS is utilized, dividing it into four phenotypes: phenotype A (hyperandrogenism, ovulatory dysfunction, and polycystic ovarian morphology); phenotype B (hyperandrogenism and ovulatory dysfunction); phenotype C (hyperandrogenism and polycystic ovarian morphology); and phenotype D (ovulatory dysfunction and polycystic ovarian morphology).^(6)^

The treatment of PCOS aims to correct the metabolic alterations caused by hormonal imbalance. Its primary goal is to improve the patient's fertility, reduce the presence of hirsutism and/or alopecia, and provide endometrial protection to prevent endometrial cancer. Therapy involves implementing lifestyle changes, sometimes accompanied by medication, and managing obesity. Additionally, long-term monitoring and control of type 2 diabetes mellitus, hypertension, and cardiovascular disease risk should be ensured.^(7)^

Given the wide range of clinical manifestations and complications caused by this condition, efforts have been made to establish the etiological mechanism that underlies PCOS.^(8)^ Current literature suggests that this disease arises from a combination of genetic, epigenetic, endocrine, metabolic, and environmental factors that predispose patients differently. In this regard, environmental factors have been shown to play a significant role in the development of this complex disorder in recent years, particularly those compounds categorized as Endocrine-Disrupting Chemicals (EDCs).^(8,9)^

EDCs constitute a broad and diverse group of molecules capable of interfering with the endocrine system and disrupting the proper function of hormones through three mechanisms. Firstly, via mimicry, EDs can mimic a hormone and induce overstimulation in its target tissue. Secondly, through antagonistic action, EDs bind to hormone receptors and prevent the generation of an action or response. Lastly, interference or blocking mechanism, wherein EDs act directly on the hormone or its receptor, triggering inadequate or no endocrine signal.^(10,11)^

These pollutants can be naturally occurring, originating from animals or plants, or as byproducts of various manufacturing processes of human consumer products.^(12)^ These compounds enter the body in small doses through various sources, such as drinking water, air, medications, food, etc.^(12,13)^ It is estimated that there are around more than 1000 chemicals that act as DE. Within this wide range of substances, we find pharmaceutical products, pesticides, fungicides, industrial chemicals, plasticizers, nonylphenols, metals, dioxins, bisphenols, and polychlorinated, among others.^(14,15)^

Exposure to EDCs is widespread among living beings, as these compounds are extensively utilized and present in various everyday products, including plastic bottles, detergents, foods, toys, pesticides, metals, and cosmetics.^(16,17)^ Consequently, exposure to various EDs in daily life is on the rise, posing a significant concern due to their toxic nature.^(18)^ Thus, the systematic review aimed to summarize the available evidence of the association between endocrine disrupters and PCOS.

## Methods

A systematic review was conducted to evaluate the association between exposure to endocrine-disrupting chemicals (EDCs) and PCOS. The review adhered to the methodological standards of the PRISMA 2020 statement.^(19)^ The protocol for this review was registered in PROSPERO, # CRD420261328419.

### Eligibility criteria

#### Inclusion criteria

Studies were included if they met the following criteria: i) Evaluated the association between PCOS and exposure to endocrine-disrupting chemical; ii) Used observational epidemiological designs, including: Cohort, Case-control, and Cross-sectional studies.

#### Exclusion criteria

Studies were excluded if they met any of the following conditions: i) Included women with PCOS and other coexisting diseases that could confound the association with EDC exposure; ii) Were *in silico, in vitro*, animal, or other non-human experimental models; iii) Were non-primary sources, including letters, conference abstracts, editorials, book chapters, and review articles; iv) Did not directly measure exposure to endocrine-disrupting chemicals or did not analyze their relationship with PCOS; v) Were published in languages other than English or Spanish or lacked accessible full text.

### Search strategy

A comprehensive literature search was performed in four electronic databases: PubMed/MEDLINE, Scopus, ScienceDirect, and LILACS, covering studies published between 2013 to 2025. An initial exploratory search in PubMed was conducted to identify relevant MeSH terms, keywords, and synonyms associated with PCOS and endocrine disruptors. This preliminary step helped refine the final search strategy, which was subsequently applied across all databases (Supplementary Material Table 1S).

The search strategy was carried out using the following keywords: Polycystic Ovary Syndrome, Ovary Syndrome Polycystic, Syndrome Polycystic Ovary, Stein-Leventhal Syndrome, Sclerocystic Ovarian Degeneration, Sclerocystic Ovary Syndrome, Polycystic Ovarian Syndrome, Sclerocystic Ovaries, PCOS, Endocrine Disruptors, Endocrine Disrupting Chemicals, Triclosan, Bisphenol A, EDCs. The update of the search was performed on November 25, 2025.

### Methodological quality assessment

Two reviewers independently evaluated the risk of bias in the included studies using the Joanna Briggs Institute (JBI) instrument.^(20)^ This method assigns a numerical value based on the study type, with a maximum score of 11 for cohort studies, 10 for case-control studies, and 8 for cross-sectional studies. No specific cutoff point was established; thus, a higher score denoted a higher methodological quality of the study.

### Data extraction and analysis

Data extraction was carried out independently by two reviewers using previously standardized templates designed for this review.^(21)^ Through this process, information was systematically obtained regarding study characteristics such as author, year of publication, study design, and country of origin as well as participant characteristics, including age of the women, biological matrix analyzed, diagnostic criteria used for PCOS, type of endocrine disruptor assessed, and its measured concentration. Additionally, reviewers extracted the reported outcomes, including effect estimates such as odds ratios or risk ratios with their corresponding 95% confidence intervals, along with the main conclusions of each study. Disagreements were resolved through consensus, in which a third reviewer participated. Due to the significant heterogeneity observed across the studies, the data compiled in this systematic review were not conducive to meta-analysis. Consequently, we opted for a descriptive presentation of the findings and conducted a narrative synthesis of the evidence.

## Results

### Selection of studies

**Figure 1:**
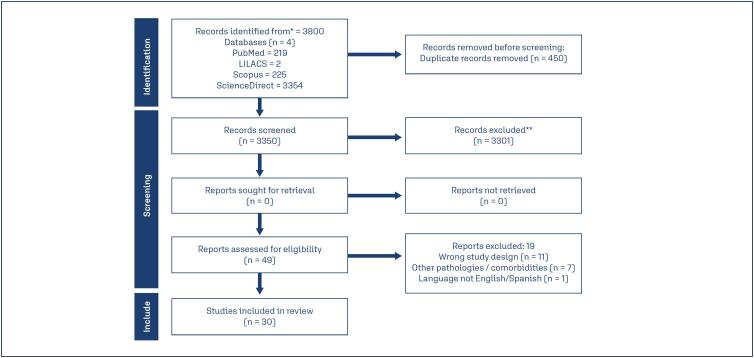
PRISMA flowchart describing the selection of studies

Initially, 3,800 publications were identified during the search procedure. Following the abstract review, 450 studies were excluded for failing to meet the inclusion criteria. This left 49 studies as potential candidates, which were then subjected to full-text review. Ultimately, 30 studies met the inclusion criteria and were selected for analysis, as depicted in figure 1.

### Characteristics of the selected studies

The selected studies^(2,22-41)^ were published between 2013 and 2025 in English, and were carried out in China (nine),^(2,26,28,29,31,38,40-42)^ Turkey (three),^(24,32,33)^ United States (three),^(23,35,43)^ India (two),^(44,45)^ Iran(two),^(25,27)^ Serbia (two),^(46,47)^ Poland (three),^(30,39,48)^ Denmark,^(49)^ Saudi Arabia,^(37)^ Czech Republic,^(34)^ Slovakia,^(36)^ Sweden^(50)^ and Italy.^(22)^ According to the type of study, six Cohort,^(24,33,35,43,49,50)^ nineteen cases and controls^(2,22,23,25,27,28,31,32,34,36-42,44,46,48)^ and five cross-sectional.^(26,29,30,45,47)^ The sample size varied according to the type of study, 124 to 24581 for cohort,^(24,33,35,43,49,50)^ 29 to 943,^(2,22,23,25,27,28,31,32,34,37-43,45,47,50)^ and 40 – 304 for cross-sectional.^(26,29,30,45,47)^ and the age of the patient had a range between 13 and 45 years. Diagnostic scales used for diagnosis in study participants included Rotterdam, modified Rotterdam, National Institutes of Health Criteria for PCOS, and Androgen Excess and PCOS Criteria. The characteristics of these studies are described in chart 1.

**Chart 1:** The characteristics of included studies

Author	Country	Type of study	Sample size	Women's age (year)	Diagnostic method
Tarantino et al. (2013)^(22)^	Italy	Cases and controls	Caso:40 Controls:20	Cases: 26.2±3.9 Controls: 27.7 ± 6.8	Rotterdam criteria
Vagi et al. (2014)^(23)^	United States	Cases and controls	Cases: 30 Controls:50	Cases: 29.7±3.2 Controls: 30.6±2.9	1990 National Institutes of Health Criteria for PCOS
Akın et al. (2015)^(24)^	Turkey	Cohort	Cases: 112 Controls: 61	13-19	Modified Rotterdam Criteria
Vahedi et al. (2016)^(25)^	Iran	Cases and controls	Caso: 62 Controls:62	Cases: 29.24±3.11 Controls: 28.56±3.29	Rotterdam criteria
Zhou et al. (2016)^(26)^	China	Cross-sectional	268	Mean: 27	Rotterdam criteria
Hossein et al. (2017)^(27)^	Iran	Cases and controls	Cases: 51 Control: 51	Cases: 29.80 ± 7.02 Controls: 32.96±5.58	Rotterdam criteria
Jin et al. (2019)^(28)^	China	Cases and controls	Cases: 56 Control: 51	20 y 45	Rotterdam criteria
Ye et al. (2018)^(29)^	China	Cross-sectional	296	18–45	Rotterdam criteria
Konieczna et al. (2018)^(30)^	Poland	Cross-sectional	304	18-40	Criteria of the Androgen Excess and PCOS Society
Gu et al. (2019)^(31)^	China	Cases and controls	Cases:40 Controls:83	Cases: 30.5±3.6 Controls: 29.8± 3.2	Rotterdam criteria
Akgül et al. (2019)^(32)^	Turkey	Cases and controls	Cases:62 Controls:33	Cases: 15.62 ± 1.29 Controls: 16.04 ± 1.59	Rotterdam criteria
Akin et al. (2020)^(33)^	Turkey	Cohort	Cases: 63 Controls: 61	13-19	Modified Rotterdam Criteria
Luo et al. (2020)^(2)^	China	Cases and controls	Cases: 119 Controls:238	Cases: 21.5±1.5 to 21.7±3.2 Controls: 22.3±16.3	Rotterdam criteria
Šimková et al. (2020)^(34)^	Czech Republic	Cases and controls	Cases: 19 Controls:20	Cases: 28.9 ± 7.4 Obese PCOS: 29.5±5.8 Controls: 29.9±6.4	National Institutes of Health Criteria for PCOS
Kim et al. (2021)^(35)^	United States	Cohort	251	18 a 44	Rotterdam Criteria
Lazúrová et al. (2021)^(36)^	Slovakia	Cases and controls	Caso: 86 Controls:32	Cases: 28.5±5.1 Controls: 24.9±4.4	Rotterdam criteria
Al-Saleh (2022)^(37)^	Saudi Arabia	Cases and controls	Cases: 82 Controls: 359	Cases: 31.63± 4.82 Controls: 32.71±4.98	Rotterdam criteria
Zhan et al. (2023)^(40)^	China	Cases and controls	Cases:366 Controls: 577	Cases: 28.0 (26.0–32.0) Control: 28.0 (26.0–32.0)	Rotterdam criteria
Zhang et al. (2023)^(38)^	China	Cases and controls	Cases of PCOS: 96 PCOS: 96 Controls:370	≤30 and >30	Rotterdam criteria
Majewska et al. (2024)^(39)^	Poland	Cases and controls	Cases:135 Controls:104	Cases: 27±5.3 Controls: 32.7±6.4	Androgen Excess and PCOS Society
Milankov et al. (2023)^(47)^	Serbia	Cross-sectional	60	26.15 ± 5.20 (range 17–40)	Rotterdam criteria
Guo et al. (2017)^(41)^	China	Case-control	Cases: 84 Controls: 94	Median age: Cases 28 (20–35) Controls 29 (22–35)	Rotterdam criteria
Kawa et al. (2019)^(44)^	India	Case–control	Cases: 49 Controls: 39	Mean age: Cases 23.7 ± 4.5 Controls 22.2 ± 3.0	Rotterdam criteria (2003)
Jurewicz et al. (2021)^(48)^	Poland	Case–control	PCOS: 199 Controls: 158	18–40 (no significant age differences reported)	AES & PCOS Society criteria
Liang et al. (2022)^(42)^	China	Case–control	Cases: 369 Controls: 441	Cases: 28.80 ± 3.39 Controls: 28.97 ± 2.39	Revised 2003 Rotterdam criteria
Milanović et al. (2020)^(46)^	Serbia	Case–control	PCOS: 29	25.76 ± 4.91	Rotterdam criteria
Tøttenborg et al. (2025)^(49)^	Denmark	Cohort	21,619	Median start age 22; median end age 44	National patient registry ICD-8/ICD-10 codes
Hammarstrand et al. (2021)^(50)^	Sweden	Cohort	24,581 women	18-44	National patient registry diagnostic codes for PCOS, leiomyoma, and endometriosis
Wang et al. (2025)^(43)^	United States	Cohort	322 mother–daughter pairs	Mothers: mean 32.6; Daughters: mid-to-late adolescence	PCOS diagnosis: self-reported PCOS or probable PCOS based on ovulatory dysfunction + clinical/biochemical hyperandrogenism (2023 PCOS Guideline)
Patel et al. (2024)^(45)^	India	Cross-sectional	40 women (25 urban, 15 rural)	17–40 years	PCOS diagnosed using Rotterdam 2003 criteria (oligo/anovulation, hyperandrogenism, and/or polycystic ovarian morphology)

PCOS: Polycystic Ovary Syndrome; PCO: polycystic ovary

### Methodological quality of the included studies

**Figure 2:**
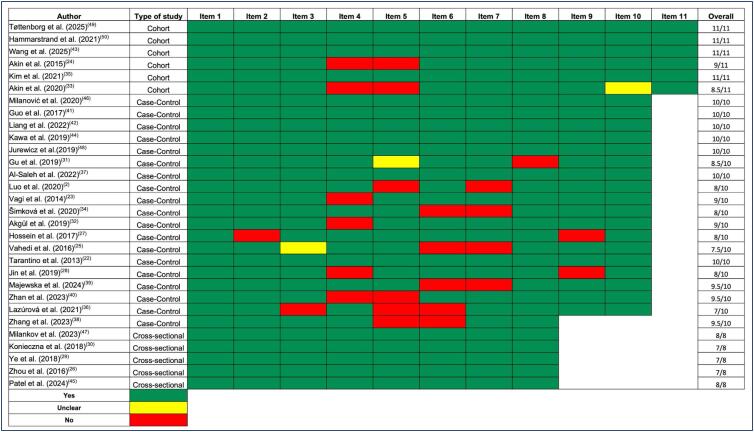
The methodological quality of the included studies by design.

Utilizing the JBI criteria, Cohort studies achieved an average score of 10.3 ± 1.17, spanning from 8.5 to 911.0. In comparison, Case-Control studies attained a mean score of 9.0 ± 01,03, with a range of 7.0 to 10, while Cross-Sectional studies scored 7.4 ± 0.55, varying from 7 to 8. The results of the methodological quality of the studies are shown in figure 2.

### EDCs and PCOS

PCOS is a complex endocrine disorder affecting women of reproductive age, characterized by symptoms such as irregular menstrual cycles, hyperandrogenism, and polycystic ovaries. Recent research has increasingly focused on the role of environmental factors in the etiology of PCOS, particularly the impact of endocrine-disrupting chemicals that can interfere with hormone function.^(9,50,51)^Chart 2 summarizes the evidence on ECDS exposure and PCOS.

**Chart 2:** Summary of evidence on EDCs and PCOS

Author (Year)	Sample	Disrupter	Concentration	Outcome	Conclusion
Tarantino et al. (2013)^(22)^	Blood	BPA	Cases: 0.7 (0.1-0.6) ng/mL Control: 0.1 (0.1-0.6) ng/mL	BPA concentrations in PCOS were associated with higher grades of insulin resistance, hepatic steatosis, androgen index, and inflammation (p<0.05).	This study showed the role of environmental exposure to bisphenol A in PCOS. Also, described the association between BPA exposure, inflammation, liver-pleen axis, and hyperandrogenism in the PCOS setting.
Vagi et al. (2014)^(23)^	Blood and urine	PCBs OCPs PBDEs PFCs. PAEs metabolites BPA	Cases: PCBs: 0.7 to 38.6 pg/L serum PBDEs: 3.3 to 144.5 pg/L serum OPCs: 16 to 1217.5 PFC: 1.1 to8.2 pg/L serum PAEs: 2.4 to 103.7 μg/L urine BPA: 1.6 μg/L urine Controls: PCBs: 1.1 to 47.2 pg/L serum PBDEs: 6.0 to 148.6 pg/L serum OPCs: 16.1 to 1397.2 PFC: 0.7 to 4.9 pg/L serum PAEs: 3.0 to 138.3 μg/L urine BPA: 2.1 μg/L urine	Patients with PCOS showed an association with levels of perfluorooctanoate and perfluorooctane sulfonate (adjusted oRs = 5.8–6.9, p < 0.05), as well as concentrations of MBzP and MBP (adjusted oRs = 0.14–0.25, p < 0.05).	PCOS cases were associated with higher serum concentrations of two PFCs and lower concentration of PAEs metabolites.
Akın et al. (2015)^(24)^	Blood	BPA	Control: 0.8 (0.6-0.9) ng/mL PCOS: 1.1 (1.0-1.2) ng/mL	Serum BPA levels were significantly increased in adolescents with PCOS compared to the control group (p = 0.001).	BPA concentrations were correlated with androgen levels but not with obesity or metabolic parameters.
Vahedi et al. (2016) ^(25)^	Blood	BPA	Cases: 0.16 ± 0.04 ng/mL Control: 0.48 ± 0.08 ng/mL	The concentration of BPA in the case group was significantly higher than that in the control group (P < 0,001).	In BPA-exposed PCOS women, BPA levels were higher than in healthy women, and this difference may be the cause of significant differences in levels of triglyceride, cholesterol, thyroid hormone, and luteinizing hormone/follicle stimulating hormone ratio.
Zhou et al. (2016)^(26)^	Urine	BPA	*2.35 ng/mL	BP-Creatinine was associated with a significant decrease of 0.34 in AFC (β = −0.34, 95% CI = −0.60, −0.08; p = 0.01). 0,34 in (−0,34, IC 95 % = −0,60, −0,08, p = 0,01).	This study suggests that in women with PCOS, BPA may affect ovarian follicles and, therefore, reduce ovarian reserve.
Hossein et al. (2017)^(27)^	Urine	BPA	Cases: 3.34±2.63 ng/mL Control: 1.43±1.57 ng/mL	OR:1.53 (95% CI: 1.14-2.05, p = 0.004).	BPA could play a major role in the etiology of PCOS.
Jin et al. (2019)^(28)^	Follicular fluid	DEHP	Cases: 1.68 (1.27-2.13) ng/mL Control: 1.21 (1.061.38) ng/mL	DEHP levels in the follicular fluid were significantly higher in women with PCOS than in controls (p<0.05).	PCOS patients are exposed to increased levels of DEHP in follicles, which may be associated with pregnancy loss following in vitro fertilization.
Ye et al. (2018)^(29)^	Urine	Triclosan	PCOS†: 0.45 (0.25-1.54) ng/mL Non-PCOS†: 0.37 (0.14-0.98) ng/mL	The highest tertile of TCS levels was associated with an increased odd of PCOS (OR 2.12; 95%: 1.12 to 3.99). After adjusting for potential confounders, the significant association remained (OR 1.99, 95% CI 1.05 to 3.79).	TCS exposure was significantly associated with PCOS in a Chinese population.
Konieczna et al. (2018)^(30)^	Blood	BPA	PCOS: 0.202 ng/mL [0.150-0.255] Without PICOS: 0.154 ng/mL [0.106 0.201].	PCOS women were correlated positively with serum total testosterone (R = 0.285, P = 0.004) and the free androgen index (R = 0.196, P = 0.049).	These results showed the potential role of BPA in the pathogenesis of ovarian hyperandrogenism.
Gu et al. (2019)^(31)^	Urine	BPA TCS HMS BP-3 OC	BPA: 0.01 to 161 μg/L TCS: 0.15–53.6 μg/L HMS: 0.04–8.21 μg/L BP-3: 0.57–450 μg/L OC: 0.09–42.8 μg/L	A statistically significant association between OC and PCOS was observed (p = 0.029) when BMI was ≥ 24.	There were no significant relationships between PCOS and urinary BPA, TCS, BP-3, HMS, and OC levels, respectively, in the overall case-control group. However, urinary OC concentrations and PCOS were positively associated with women with BMI ≥ 24.
Akgül et al. (2019)^(32)^	Blood	BPA DEHP MEHP	Cases: BPA 15.89 ± 1.16 μg/g Creatinine DEHP: 0.40 ± 0.24 μg/g Creatinine MEHP: 0.13 ± 0.23 μg/g Creatinine Control: BPA: 7.3 ± 1.38 μg/g creatinine DEHP: 0.49 ± 0.27 μg/g creatinine MEHP: 0.14 ± 0.3 μg/g creatinine	BPA was significantly correlated with polycystic morphology on ultrasound (P= 0.038). No association were found between, DEHP, MEHP and PCOS.	Significant relationship between urinary BPA concentrations in adolescents with PCOS.
Akin et al. (2020)^(33)^	Blood	DEHP MEHP	Control: 2.71 (2.52-2.90) µg/mL PCOS: 2.62 (2.50-2.75) µg/mL	A correlation was found between: MEHP or DEHP and Glucose (p<0.05), insulin (p<0.05), and HOMA-IR(p<0.05). Triglyceride levels only was correlated with MEHP.	Serum DEHP and MEHP levels in adolescents with PCOS were not different from controls, but both were correlate with insulin resistance and metabolic disturbances in patients with PCOS.
Luo et al. (2020)^(2)^	Blood	BPA PBDE PAE	†Controls: BPA: 4.8 ± 2.9 ng/mL PBDEs:126.3 ±48.9 ng/mL PAEs: 41.5±30.1 ng/mL †Cases: BPA: 6.42 ± 3.74 ng/mL PBDEs: 135.5 ±46.2 ng/mL PAEs: 58.5±36.5 ng/mL	Patients with PCOS had a higher BPA concentration compared to the control group (P = 0.01). Also, patients with PCOS had higher PAE levels than controls (P = 0.03).	TT genotype of UGT2B7H268Y was associated with an increased risk of PCOS, through a decreased clearance of BPA, and PAEs and androgen in patients with PCOS.
Šimková et al. (2020)^(34)^	Blood	BPA BPS Paraben= ∑ Methyl paraben, Propyl paraben, Ethyl paraben, Butyl paraben, Benzyl paraben	Control‡: BPA: 0.129 (0.065- 0.211) nmol/L BPS: 0 (0- 0.078) nmol/L ∑parabens: 0 (0-0.391) nmol/L PCOS with normal weight‡: BPA: 0.282 (0.129-0.356) nmol/L BPS: 0 (0-0.155) nmol/L ∑parabens: 0.488 (0- 2.85) nmol/L PCOS obese‡: BPA: 0.129 (0.129-0.192) nmol/L BPS: 0 (0-0.155) nmol/L ∑parabens: 0 (0-0) nmol/L	BPA exposure was significantly higher in normal-weight PCOS women than in healthy controls (p=0.042). There was no difference between normal-weight and obese PCOS women (p>0.05).	The higher levels of BPA in PCOS patients can be related to their role in the etiopathology of PCOS.
Kim et al. (2021)^(35)^	Blood	Cadmium	0.30* (0.19–0.43) µg/L	For each 0.1 µg/L increase in blood cadmium levels, was associated with an 18 % higher probability of a mild PCOS-phenotype (RR 1.18; 95 % CI 1.06- 1.31).	Cadmium was associated with endocrine features central to PCOS (testosterone, SHBG and AMH).
Lazúrová et al. (2021)^(36)^	Urine	BPA	Cases: 6.1 ± 0.9 μg/g creatinine Control: 1.6 ± 0.6 μg/g creatinine	PCOS women had significantly higher urine-BPA as compared with control group (p = 0.0001).	Urine-BPA was negatively associated with serum ovarian estrogen and androgen concentrations, which indicates that BPA may exert a possible suppressive effect on ovarian steroidogenesis.
Al-Saleh (2022)^(37)^	Urine	PAEs metabolites	Controls: MEP: 685.4 ±1269.1 μg/L MiBP: 68.2±60 μg/L MnBP: 178.5±244 μg/L MBzP: 2.2±4.5 μg/L MECPP: 39.9±61.4 μg/L MEHHP: 16.8±40.0 μg/L MEOHP: 26.4±43.0 μg/L MEHP: 18.8±20.7 μg/L ∑4DEHP: 0.3±0.5 μmol/L Cases: MEP: 774.0 ±1248.6 μg/L MiBP: 75.1±59.4 μg/L MnBP: 196.3±225.6 μg/L MBzP: 2.2±2.0 μg/L MECPP: 60.6.9±125.4 μg/L MEHHP: 19.5±29.8 μg/L MEOHP: 41.5±90.9 μg/L MEHP: 27.4±62.1 μg/L ∑4DEHP: 0.5±0.1 μmol/L	Higher odds of PCOS were associated with levels of MECPP (OR: 1.386; 95% CI: 1.033-1.860), MEOHP (OR: 1.411; 95% CI: 1.055-1.885) and, ∑4DEHP (OR: 1.405; 95% CI: 1.025-1.925).	The sum of the four di (2-ethylhexyl) phthalate ∑4DEHP) and their metabolites (MEP and (MECPP) may contribute to PCOS.
Zhan et al. (2023)^(40)^	Blood	PFAS	†0.04-8.52 %	PFAS were associated with an increased prevalence of PCOS, odds ratio of 1.20 [95% CI: 1.06-1.37].	Exposure to a PFAS mixture was associated with an increased prevalence of PCOS in women of reproductive age
Zhang et al. (2023)^(38)^	Urine	Phthalate metabolites	Control† MEP: 11.35 (5.44–25.87) ng/mL MiBP: 19.14 (13.09–30.18) ng/mL MBP: 149.58 (83.61–249.01) ng/mL MBzP: 0.07 (0.04–0.12) ng/mL MEHHP: 8.95(6.16–13.67) ng/mL MECPP: 14.26(9.74–20.86) ng/mL MEOHP: 6.94(4.76–10.44) ng/mL MEHP: 3.97(2.43–6.75) ng/mL ∑DEHP: 0.12(0.08–0.19) ng/mL PCO† MEP: 12.19 (5.93–26.87) ng/mL MiBP: 25.33 (13.81–34.40) ng/mL MBP: 161.69 (94.84–266.17) ng/mL MBzP: 0.09 (0.06–0.16) ng/mL MEHHP: 9.97 (6.65–15.28) ng/mL MECPP: 15.23 (11.18–21.30) ng/mL MEOHP: 7.22 (4.83–10.12) ng/mL MEHP: 4.05 (2.26–6.56) ng/mL ∑DEHP: 0.12(0.09–0.21) ng/mL PCOS† MEP: 11.05 (6.04–23.89) ng/mL MiBP: 21.39 (13.22–44.23) ng/mL MBP: 169.37 (78.84–295.96) ng/mL MBzP: 0.09 (0.06–0.18) ng/mL MEHHP: 9.62 (7.06–14.66) ng/mL MECPP: 15.24 (10.72–21.85) ng/mL MEOHP: 7.48 (5.15–11.17) ng/mL MEHP: 3.82 (2.53–7.67) ng/mL ∑DEHP: 0.14 (0.09–0.23) ng/mL	MiBP, MBzP and ∑DEHP con- centrations was associated with increased prevalence of PCO, and the adjusted oRs (aORs) were 1.32 (95% CI: 1.02- 1.72), 1.40 (95% CI: 1.10-1.78) and 1.35 (95% CI: 1.01- 1.80), respectively. The higher prevalence of PCOS were estimated for one ln-unit increase in MBzP (aOR= 1.51,95% CI: 1.19-1.91), MEHP (aOR= 1.18, 95% CI: 1.00- 1.40) and ∑DEHP (aOR= 1.44, 95% CI: 1.08-1.92) concentrations.	These findings suggest a potential role for phthalate exposures, both individually and in mixtures, in the development of PCO and PCOS.
Majewska et al. (2024)^(39)^	Blood	BPA analogues	†Control: BFE: 0.039 (0.011-0.152) ng/mL BPC: 0.033 (0.018- 0.813) ng/mL BPG: 0.054 (0.021- 0.268) ng/mL BPM: 0.058 (0.033- 0.123) ng/mL BPP: 0.047 (0.017-0.146) ng/mL BPZ: 0.092 (0.025-0.193) ng/mL BPFL: 0.010 (0.006-0.129) ng/mL BPBP: 0.030 (0.011- 0.614) ng/mL †Cases: BFE: 0.037 (0.013- 0.185)ng/mL BPC: 0.029 (0.009-0.890) ng/mL BPG: 0.052 (0.016- 0.278) ng/mL BPM: 0.067 (0.013-0.115) ng/mL BPP: 0.049 (0.009- 0.129) ng/mL BPZ: 0.084 (0.027- 0.157) ng/mL BPFL: 0.011 (0.008-0.127) ng/mL BPBP: 0.031 (0.009- 0.754) ng/mL	There was not association between levels of BPA analogues (BPE, BPC, BPG, BPM, BPP, BPZ, BPFL, and BPBP) and the diagnosis of PCOS.	This study didn´t find differences in serum BPE, BPC, BPG, BPM, BPP, BPZ, BPFL, and BPBP levels in women diagnosed with PCOS compared to healthy controls.
Milankov et al. (2023)^(47)^	Urine	Phthalates (10 metabolites: MMP, MEP, MBP, MPP, MCHP, MOP, MEHP…)	MMP: 1.55–35.10 MEP: 4.90–20.04 MPP: 1.05–3.19 MCHP: 10.70 MOP: 2.33–5.10 MEHP: 0.51–18.26 Any metabolite detected in: 51.7% of women (Undetected: MiAP, MnAP, MBzP)	Women with PCOS showed detectable urinary phthalate metabolites in more than half of the samples, and higher total phthalate concentrations were significantly associated with adverse metabolic parameters, including increased BMI, waist circumference, visceral adiposity indices (LAP and VAI), fasting glucose, and HOMA-IR. Phthalate exposure was also linked to dyslipidemia, reflected in higher total cholesterol, triglycerides, LDL levels, and TC/HDL ratios. Notably, mono-methyl-phthalate (MMP) demonstrated the strongest correlations, being associated with impaired glucose metabolism, insulin resistance, and elevated testosterone levels, suggesting a combined metabolic and androgenic disruption in PCOS.	Phthalate exposure—especially MMP—may contribute to metabolic dysfunction in women with PCOS, including obesity, insulin resistance, dyslipidemia, visceral adiposity, and increased testosterone levels.
Guo et al. (2017)^(41)^	Serum	OCPs (β-HCH, γ-HCH, p,p′-DDT, o,p′-DDT, p,p′-DDD, p,p′-DDE)	p,p′-DDT: PCOS 0.77 ng/mL vs. controls 0.58 ng/mL (p = 0.016)	Women with PCOS exhibited significantly higher serum levels of p,p′-DDT and o,p′-DDT. Increasing o,p′-DDT levels were positively associated with higher LH/FSH ratio, elevated testosterone, increased triglycerides, and lower FSH and SHBG, suggesting endocrine-metabolic disruption.	Elevated levels of o,p′-DDT and p,p′-DDT may contribute to hormonal imbalance, hyperandrogenism, and metabolic disturbances in PCOS, supporting a potential pathogenic role for OCP exposure.
Kawa et al. (2019)^(44)^	Serum	Bisphenol A (BPA)	PCOS: 26.4 ± 14.9 ng/mL	Women with PCOS exhibited significantly higher BPA concentrations than controls. BPA levels showed robust positive correlations with adiposity markers (BMI, waist circumference, WHR), hyperandrogenism (testosterone), glycemic abnormalities (fasting, 1-h, and 2-h glucose), dyslipidemia (cholesterol and triglycerides), and insulin resistance indices (fasting insulin, HOMA-IR), along with a negative association with QUICKI. Hematological parameters such as hematocrit and MCV were also positively associated.	BPA exposure appears to contribute to the hormonal, metabolic, and hematological alterations characteristic of PCOS, supporting its role as a potential environmental driver of PCOS pathogenesis.
Jurewicz et al. (2021)^(48)^	Serum	BPA, BPS, BPF	BPA: No significant difference between PCOS and controls. BPS: PCOS: 0.14 ng/mL vs. controls: 0.08 ng/mL (p = 0.023). BPF: No significant difference.	Women with PCOS showed significantly higher BPS concentrations, while BPA and BPF did not differ between groups. BPA showed negative correlations with HOMA-IR and testosterone. Logistic regression indicated increased odds of PCOS among women in the lowest tertile of BPS exposure	BPS, but not BPA or BPF, was associated with PCOS risk. BPA may relate to metabolic and hormonal alterations, but no consistent patterns were found for BPS or BPF.
Liang et al. (2022)^(42)^	Fasting blood	Pb, Hg, As, Ba, Cd	Concentrations significantly higher in PCOS: Pb: GM 29.34 μg/L vs. 24.51 μg/L (p < 0.001) As: GM 2.34 μg/L vs. 1.74 μg/L (p < 0.001) Ba: GM 29.46 μg/L vs. 23.93 μg/L (p = 0.002) No differences: Hg, Cd	Higher levels of Pb, As, and Ba were associated with increased PCOS risk. For each ln-unit increase: Pb: aOR 1.83 (1.35–2.48); As: aOR 2.49 (1.86–3.33); Ba: aOR 1.20 (1.04–1.39). Women in the highest tertiles of Pb, As, and Ba had significantly elevated odds of PCOS. BKMR showed a positive joint effect of the five-metal mixture on PCOS risk, with As (PIP = 100%) and Pb (PIP = 67.44%) contributing most	Exposure to toxic metals—particularly As, Pb, and Ba—was significantly associated with increased PCOS risk, both individually and as part of a metal mixture. Findings support a potential endocrine-disrupting role of these metals in PCOS pathophysiology.
Milanović et al. (2020)^(46)^	Urine	BPA	Detected in 48.3% of PCOS women; levels ranged 3.01–39.09 µg/g creatinine	BPA exposure was associated with significantly higher WtHR (p = 0.046), moderate increases in waist circumference and BMI, higher odds of central obesity (WC > 80 cm and WtHR > 0.5), elevated insulin (p = 0.038), and trends toward higher HOMA-IR and lower HDL.	BPA exposure was associated with significantly higher WtHR (p = 0.046), moderate increases in waist circumference and BMI, higher odds of central obesity (WC > 80 cm and WtHR > 0.5), elevated insulin (p = 0.038), and trends toward higher HOMA-IR and lower HDL.
Tøttenborg et al. (2025)^(49)^	Indoor air (residential exposure)	Lower-chlorinated PCBs (LC-PCBs)	Median exposure 56.3 PCByear (range 0–43,099); PCBtotal in contaminated apartments: 871–1298 ng/m³	No association between PCB exposure and incidence of UL, endometriosis, or PCOS. Adjusted HRs ∼1.00 for all outcomes.	No association between PCB exposure and incidence of UL, endometriosis, or PCOS. Adjusted HRs ∼1.00 for all outcomes.
Hammarstrand et al. (2021)^(50)^	Population exposure via drinking water	PFAS (PFOS, PFOA, PFHxS, PFNA)	Exposure categories based on municipal water PFAS concentrations	Higher PFAS exposure was not associated with increased risk of PCOS, but was associated with higher incidence of uterine leiomyoma and endometriosis in certain exposure groups.	PFAS-contaminated drinking water did not show a significant association with PCOS risk, but may increase the risk for other gynecologic conditions (leiomyoma, endometriosis).
Wang et al. (2025)^(43)^	Maternal plasma during early pregnancy; offspring clinical data	PFAS (PFHxS, PFOS, PFOA, PFNA, MeFOSAA, EtFOSAA)	Median PFAS levels: PFOS 25.3 ng/mL; PFOA 5.4 ng/mL; PFHxS 2.2 ng/mL; PFNA 0.7 ng/mL; MeFOSAA 1.8 ng/mL; EtFOSAA 1.1 ng/mL	Higher maternal EtFOSAA concentrations were associated with increased odds of self-reported PCOS in daughters (OR 2.66, 95% CI 1.18–5.99). Maternal PFNA was associated with moderate-to-severe acne in daughters (OR 2.33, 95% CI 1.09–4.99). No significant associations were found for irregular menstrual cycles or hirsutism. PFAS mixture showed no association with PCOS or related traits.	Prenatal exposure to specific PFAS, particularly EtFOSAA, may increase the risk of PCOS in offspring during adolescence. However, the PFAS mixture overall was not associated with PCOS, indicating compound-specific rather than mixture-driven effects.
Patel et al. (2024)^(45)^	Serum from 40 PCOS women (urban n=25; rural n=15)	BPA, MEHP, DEHP	BPA: 118.95 vs. 33.96 ng/mL; MEHP: 23.86 vs. 20.11 μg/mL; DEHP: 9.08 vs. 2.18 μg/mL (urban vs rural)	Urban PCOS: significantly higher BPA and DEHP levels; MEHP slightly elevated. BPA showed negative correlation with TSH. Rural PCOS: DEHP strongly associated with increased estradiol and decreased PRL and DHEAS; MEHP negatively associated with DHEAS.	BPA and phthalate exposure differs sharply between urban and rural environments. DEHP displays strong hormone-disrupting effects, particularly in rural women. Environmental context modifies the endocrine impact of EDCs in PCOS.

* Median

† Geometric mean

‡ Medians with lower and upper quartiles

AMH: Anti Müllerian hormone; SHBG: Sex hormone-binding globulin; DEHP: Di-2-ethylhexyl phthalate; MEHP: 2-ethylhexyl) phthalate; HOMA-IR:homeostasis model assessment-insulin resistance; BPA: Bisphenol A; TCS: Triclosan; HSM: homomethyl salicylate; BP-3: benzophenone-3; OC: octocrylene; BMI: Body mass index; MEP: monoethyl phthalate; MnBP: mono-n-butyl phthalate; MiBP: mono-iso-butyl phthalate; MBzP: mono-benzyl phthalate; MEHP: mono-(2-ethylhexyl) phthalate; MEHHP: mono-(2-ethyl-5-hydroxyhexyl) phthalate; MEOHP: mono-(2-ethyl-5-oxohexyl) phthalate; MECPP: mono-(2-ethyl-5-carboxypentyl) phthalate; ∑_4_DEHP: concentrations sum of MEHP, MEHHP, MECCP and MEOHP by their molecular weight of 278.34, 294.34, 292.33, and 308.33, respectively; PAEs: phthalates; PCBs: polychlorinated biphenyls; OCPs: Organochlorine pesticides; PBDEs: polybrominatediphenyl ethers; PFC: perfluorinated compounds; BPS: Bisphenol S; DEHP: Di-(2-ethylhexyl)-phthalate; MEHP: Mono-(2-ethylhexyl)-phthalate; PFAS:polyfluoroalkyl substances

### Bisphenol A

In this systematic review, BPA was found to be the EDCs most associated with PCOS.^(2,22-27,30-32,34,35,38,43,44,47,52)^ This compound was analyzed in blood and urine samples concentrations ranges were approximately 0.029 ng/mL – 26.4 ng/mL and 0.01 – 161 ng/mL, respectively. In total, 13 studies showed a positive association between PCOS and BPA of which 11 were case-control studies,^(2,22,23,25,27,32,37,45,47,49)^ one was a cohort study,^(23)^ and two was a cross-sectional study.^(29)^ In this sense, BPA is considered a xenoestrogen commonly used in industry, especially for coatings, due to its ability to withstand chemicals, it could disrupt the functioning of the endocrine system by mimicking the behavior of natural estrogen, 17-β estradiol, due to its structure like that of estradiol and diethylstilbestrol.^(46)^ This allows BPA to bind to estrogen receptors and cause effects on steroidogenesis, which may explain the conditions associated with PCOS.^(53)^ Furthermore, its widespread distribution and daily use generate constant exposure in the general population, predisposing to a higher risk of disease.^(46,53)^ On the other hand, other studies sought to determine the possible association between BPA and PCOS, considering metabolic and hormonal parameters. In this sense, Tarantino et al.,^(22)^ found a higher degree of insulin resistance, hepatic steatosis, and higher levels of androgens and degrees of inflammation. The literature describes that this endocrine disorder has an impact on lipid control, associated with obesity and hormonal imbalance in the hypothalamic-pituitary-gonadal axis, as identified by Luo et al.,^(2)^ and Vahedi et al.,^(25)^ which observed the presence of alterations in androgen levels, clinically described as a state of hyperandrogenism, as reported by Konieczna et al.^(30)^ These events are basically due to the ability of BPA to accumulate in the body, associated with its ability to mimic estradiol, which generates a disruption in the feedback of steroids at the hypothalamic-pituitary level and the steroid action at the ovarian level, thus suppressing the functions of the axis. This is reflected in a hypersecretion of circulating LH and elevated levels of FSH. This contributes to inflammatory conditions, triggering the infiltration of macrophages into adipose tissue, promoting obesity and subsequently a state of insulin resistance.^(54,55)^

Currently, it is known that BPA plays a role in the pathophysiology of PCOS and is considered an independent risk factor in fertile women, especially adolescents, as reported by Akın et al.,^(24)^ with results like those described by Akgül et al.,^(32)^ which also associated ultrasound changes suggestive of PCOS. The presence of BPA conditions causes states of hyperinsulinemia that can induce the secretion of GnRH and LH pulses, capable of causing a relative resistance of follicles to FSH and a subsequent increase in the production of the anti-Müllerian hormone, promoting a decrease in the count of antral follicles, which limits expression in the follicular fluid, causing dysregulation in estrogen production, and generating an unregulated hormonal metabolic state.^(56)^

Zhou et al.,^(26)^ described the main complications of BPA associated with PCOS, highlighting the reduction in ovarian reserve and predisposition to infertility by affecting ovarian follicles, as well as hormonal dysfunction of the ovary. BPA could alter gene expression, affecting endocrine processes such as the secretion and receptivity of gonadotropins, ovarian steroidogenesis, insulin activity, and the regulation of adipokines.^(54)^ These alterations lead to conditions such as ovulatory dysfunction, altered folliculogenesis, polycystic ovarian morphology, hyperandrogenism, hyperinsulinemia, and obesity, all of which are associated with PCOS. However, the development of this pathology is multifactorial, involving individual factors such as lifestyle, genetics, and associated diseases, as well as external conditions, including BPA, which contribute to the development of this disease.^(54)^

Kawa et al.^(44)^ conducted a case–control study in India, including 49 women with PCOS and 39 healthy controls diagnosed according to the Rotterdam 2003 criteria. The authors quantified serum bisphenol A (BPA) concentrations and evaluated their association with anthropometric, hormonal, metabolic, and hematological parameters. Women with PCOS exhibited significantly higher BPA levels compared with healthy controls (26.4 ± 14.9 vs. 18.95 ± 8.88 ng/mL; p = 0.0046). Higher BPA concentrations were positively correlated with adiposity indicators such as BMI, waist circumference, and waist–hip ratio; markers of hyperandrogenism, including total testosterone; and multiple metabolic abnormalities. These included elevated fasting, 1-hour, and 2-hour glucose levels, increased triglycerides and total cholesterol, and pronounced insulin resistance, as reflected by higher fasting insulin and HOMA-IR values, as well as lower QUICKI scores. Additionally, BPA levels showed positive associations with hematocrit and mean corpuscular volume.^(44)^

On the otherhand, Jurewicz et al.^(48)^ conducted a large case–control study in Poland including 199 women with PCOS and 158 healthy controls to evaluate serum concentrations of bisphenol A (BPA) and two structural analogues, bisphenol S (BPS) and bisphenol F (BPF). Using high-performance liquid chromatography with tandem mass spectrometry (HPLC-MS/MS), the authors found that serum BPS concentrations were significantly higher in women with PCOS compared with controls (0.14 vs. 0.08 ng/mL; p = 0.023). In contrast, BPA and BPF levels did not differ between groups. Interestingly, within the PCOS cohort, BPA showed negative correlations with HOMA-IR and total testosterone, but none of the bisphenols correlated with serum lipids, glucose, insulin, DHEA-S, androstenedione, or FAI. Logistic regression analysis indicated that women in the lowest tertile of BPS exposure had a higher likelihood of receiving a PCOS diagnosis, even after adjusting for sociodemographic and lifestyle factors (adjusted OR 1.12; 95% CI 1.03–3.71).^(48)^

Milanović et al.^(46)^ evaluated urinary BPA exposure among 29 women with PCOS diagnosed under Rotterdam criteria. BPA was detected in 48.3% of participants, with concentrations ranging from 3.01 to 39.09 µg/g creatinine. BPA-exposed women (PCOS BPA+) exhibited significantly higher waist-to-height ratio compared with BPA− participants (p = 0.046) and showed moderately elevated waist circumference and BMI (p = 0.057 and p = 0.078). BPA+ women had markedly increased odds of central obesity, including a 6.88-fold higher risk of waist circumference >80 cm and a 4.95-fold higher likelihood of WtHR >0.5. BPA+ participants also displayed higher insulin concentrations (p = 0.038), with trends toward increased HOMA-IR and reduced HDL cholesterol. Logistic models showed elevated odds of hyperandrogenemia (OR = 3.75) among BPA-exposed women. Collectively, these findings indicate that BPA exposure in PCOS is associated with greater metabolic risk, particularly visceral adiposity, hyperinsulinemia, insulin resistance, dyslipidemia, and elevated testosterone levels.^(46)^

Finally, only two studies did not show a statistically significant relationship between BPA and PCOS. Majewska et al.,^(39)^ evaluated the possible relationship of BPA analogs with the development of this disease, starting from the hypothesis that, having a similar chemical structure, they could have a similar action. However, none were shown to be related, although their results are not conclusive and suggest the need for broader studies. On the other hand, Gu et al.,^(31)^ compared various endocrine disruptors, including BPA, but found no evidence linking it to a predisposing factor.

### Phthalates

Phthalate was the second-most studied endocrine disruptor related to PCOS. In total, five studies aimed to find a possible relationship. The samples used were mainly blood;^(31,32,37)^ however, urine^(36,46)^ and follicular fluid^(27)^ samples were also used, with concentrations ranging from 2.50-2.90 µg/mL, 27.4-62.1 µg/L, and 1.27-2.13 ng/mL, respectively. Four studies were case control,^(27,31,36,37)^ and two was a cross-sectional cohort.^(32,46)^ The authors Akin et al.,^(33)^ Zhang et al.,^(38)^ Akgül et al.,^(32)^ and Al-Saleh^(37)^ studied the relationship between phthalate and PCOS, finding a statistically significant relationship in their results.

Phthalates are chemicals widely used in the industrial manufacture of plasticizers, which provide elasticity to plastic products.^(57,58)^ They are divided into two groups according to their molecular weight: long-chain or high molecular weight phthalates, such as di(2-ethylhexyl) phthalate (DEHP) and di-iso-nonyl phthalate (DiNP); and short-chain or low molecular weight phthalates, like dimethyl phthalate (DMP) and diethyl phthalate (DEP), which are more present in personal care products, solvents, or adhesives.^(57)^

However, Akin et al.,^(33)^ studied the presence of Di-2-ethylhexyl phthalate (and its metabolite mono (2-ethylhexyl) phthalate) with the presence of typical PCOS metabolic manifestations in adolescents. These metabolites had previously been associated with a higher prevalence of PCOS in young women of reproductive age, which was related to metabolic disturbances, including insulin resistance indices and serum triglycerides. This could even accelerate the onset of puberty in girls, giving them a higher BMI due to the obesogenic effect this compound generates in the body.^(58)^

Phthalates can disrupt the development of reproductive systems, associated with the endocrine properties of these EDCs in women. These compounds engage in inhibiting the development of antral follicles, leading to a consequent decrease in their number.^(58)^ These processes can be observed through morphological changes, as described by Akgül et al.,^(32)^ who observed polycystic features in the ovaries of women with high levels of phthalates via ultrasound, showing clinical manifestations suggestive of PCOS, associated with menstrual cycle changes.

Its mechanism of action is thought to rely on its ability to bind to estradiol receptors. Although it generates weak estrogenic activity, when sustained in the body, it sensitizes the pituitary gland to GnRH secreted by the hypothalamus, causing elevated LH levels. Meanwhile, resistant estrogenic stimulation will have an inhibitory effect on pituitary FSH. Both events will contribute to developing a pathological endocrine environment in the pituitary gland of people with PCOS.^(57)^

It has also been proposed that it acts by stimulating inflammatory factors, particularly tumor necrosis factor, which contributes to inducing apoptosis in the ovary. This mechanism, by which phthalates stimulate steroidogenesis in granulosa cells, results from feedback caused by increased levels of steroidogenic enzymes. Additionally, a complex cytokine-mediated process has been associated with immature follicular development, leading to severe consequences for female fertility.^(59)^

Jin et al.,^(28)^ unlike other studies, evaluated di-2-ethylhexyl phthalate levels in follicular fluid and found higher levels in women with PCOS, potentially associated with pregnancy loss after in vitro fertilization. This state is closely related to estrogen deficiency, menstrual cycle disturbances such as anovulatory states, and infertility.^(60,61)^

A recent cross-sectional study conducted in Serbia by Milankov et al.^(47)^ further supports the association between phthalate exposure and PCOS-related metabolic disturbances. Among 60 women with PCOS, 51.7% had detectable urinary phthalate metabolites, with MEHP and MMP being the most prevalent. The total phthalate burden was positively associated with BMI, waist circumference, WtHR, LAP, VAI, fasting glucose, and HOMA-IR, as well as with lipid abnormalities, including elevated total cholesterol, triglycerides, LDL, and TC/HDL ratios. Importantly, MMP demonstrated the strongest associations, correlating with glucose and insulin levels, markers of visceral adiposity, dyslipidemia, and increased testosterone. These findings suggest that phthalates, particularly MMP, may contribute to both metabolic and hormonal dysregulation in women with PCOS.^(47)^

### Cadmium

Cadmium was the only heavy metal linked to PCOS. Kim et al.,^(35)^ assessed serum cadmium and PCOS, associating it with the presence of phenotypic manifestations of PCOS, as well as elevated testosterone, and displaying central endocrine characteristics typical of this disease. Cadmium is considered a metalloestrogen capable of binding to the estrogen receptor, influencing alterations in androgen biosynthesis. It also acts through the pituitary gland, altering the pulsatility of gonadotropins, thereby affecting the release of gonadotropic hormones, which subsequently alter testosterone levels. This observed association may be related to cadmium's ability to bind to sex hormone-binding globulin, competing with estradiol and androgens, leading to slight increases in circulating testosterone levels, though specific mechanisms are unclear. However, considering that cadmium can also induce apoptosis in the pancreas, which manifests as a state of hyperglycemia, and its ability to cause androgen dysregulation, it leads to metabolic changes associated with PCOS.^(62,63)^

Liang et al.^(42)^ investigated the association between toxic metal exposure and PCOS in a case–control sample of 369 women with PCOS and 441 controls diagnosed using the revised Rotterdam criteria. Women with PCOS exhibited significantly higher blood concentrations of lead (29.34 vs. 24.51 μg/L; p < 0.001), arsenic (2.34 vs. 1.74 μg/L; p < 0.001), and barium (29.46 vs. 23.93 μg/L; p = 0.002), whereas mercury and cadmium did not differ between groups. Increasing exposure to Pb, As, and Ba was associated with elevated PCOS risk (aOR per ln-unit: Pb 1.83, As 2.49, Ba 1.20), and women in the highest tertiles had the greatest odds of PCOS. Bayesian Kernel Machine Regression revealed a positive joint effect of the five-metal mixture on PCOS likelihood, driven primarily by arsenic (PIP = 100%) and lead (PIP = 67.44%). Metal exposure was also linked to alterations in PCOS-related phenotypes: As was associated with higher LH and LH/FSH ratio, Ba with lower FSH, and Pb with increased fasting insulin and HOMA-IR.^(42)^

### Triclosan

Triclosan was another EDCs associated with PCOS. Ye et al.,^(29)^ described an increased risk of PCOS in patients with high levels of triclosan in their urine. However, Gu et al.,^(31)^ examined a set of EDCs, including triclosan, but did not find evidence of its relationship with PCOS. Triclosan is a compound widely used in personal care, household, pharmaceutical, veterinary, and industrial products due to its broad-spectrum antibacterial and antifungal properties.^(64)^ This EDC has a structure like anthropogenic estrogens that can activate estrogen receptors, increasing their secretion, disrupting endocrine homeostasis, and directly influencing reproductive health. Additionally, triclosan has been observed to affect the progesterone production of luteal cells and disrupt ovarian function, resulting in a state like PCOS. Although current evidence is limited and does not establish a causal relationship in women.^(64-66)^

### Other disruptors

There are other studies that have evaluated multiple EDCs and their relationship with PCOS. Among them, Gu et al.,^(31)^ evaluated BPA, TCS, HMS, BP-3, and OC, but only found a relationship in those patients with OC who had a higher BMI. There aren't enough reports on the effect of OC on the development of PCOS, but Zhang et al.^(67)^ studied the exposure to this EDC in zebrafish (*Danio rerio*) using a UV-octocrylene (OCT) filter. An apparent downregulation was observed in the ovaries, and the extent of the effects on zebrafish varied with different levels of accumulation. However, when looking at the histological changes in the ovaries, signs of estrogenic activity were shown, with activation of receptors leading to antiestrogenic and antiandrogenic activity.^(67)^

Zhan et al.,^(40)^ studied perfluoroalkyl and polyfluoroalkyl substances (PFAS) and PCOS, observing an increased prevalence of this disease in reproductive-age women with higher exposure to these hydrocarbons. PFAS are widely used in the industry for producing fire-fighting agents, cosmetics, and herbicides due to their biochemical stability and hydrophobic and oleophobic nature. These unique properties have resulted in widespread use, even in everyday applications like non-stick coatings for cookware and some clothing. The effects described by PFAS exposure include liver toxicity, reproductive disorders, neurotoxicity, and immunotoxicity. All are highly associated with hormonal homeostasis disruption.^(67,68)^

The effect of PFAS may be attribut"d to’Its impact on the distribution of sex hormones through mechanisms involving estrogen receptor activation and the transcription of specific genes associated with lipid metabolism, including cholesterol biosynthesis. On the other hand, PFAS disrupts folliculogenesis, particularly the number of follicles. These have also been implicated in actions targeting the hypothalamic-pituitary-gonadal axis, directly affecting target cells, but the precise effect on these tissues remains unclear.^(68,69)^

Vagi et al.,^(23)^ obtained similar results to study the association of several EDCs, such as PCBs, OCPs, PBDEs, PFCs, PAEs, and BPA, with PCOS. PFAS was among the most associated, but Vagi et al.,^(23)^ also identified some polychlorinated biphenyls as contributing factors. Polychlorinated biphenyls are artificial chemicals that can disrupt follicular steroidogenesis, either by mimicking hormones, altering the hormonal synthesis pattern, modulating the affinity or number of hormonal receptors, or affecting the enzymes involved in hormone secretion. By accumulating in pre-ovulatory antral follicles, these chemicals create hormonal imbalances.

In a case–control study involving 178 Chinese women, Guo et al.^(41)^ assessed serum concentrations of organochlorine pesticides (OCPs) and their association with polycystic ovary syndrome (PCOS). While no significant differences were observed between cases and controls for β-HCH, γ-HCH, p,p′-DDD or p,p′-DDE, women with PCOS exhibited significantly higher serum levels of p,p′-DDT (0.77 vs. 0.58 ng/mL; p = 0.016) and o,p′-DDT (0.53 vs. 0.44 ng/mL; p < 0.001). Increasing o,p′-DDT concentrations were positively correlated with key reproductive and metabolic features of PCOS, including higher LH/FSH ratio, elevated total testosterone and triglyceride levels, and inversely correlated with FSH and SHBG levels. The authors suggest that o,p′-DDT may exert estrogen-mimetic and steroidogenic-disrupting effects that could contribute to ovarian hyperandrogenism and metabolic alterations in PCOS. Overall, these findings strengthen the evidence that OCP exposure may play a pathogenic role in the endocrine and metabolic disturbances characteristic of PCOS, particularly in regions with historical DDT usage.^(41)^

Tøttenborg et al.^(49)^ conducted a large register-based cohort study including 21,619 Danish women living in two partially PCB-contaminated housing complexes between 1970 and 2018. Residential exposure to airborne lower-chlorinated PCBs was quantified using annual cumulative exposure (PCByear), derived from indoor air measurements and relocation histories. Over approximately 380,000 person-years, 662 incident uterine leiomyomata, 199 endometriosis cases, and 190 PCOS diagnoses were identified. Median cumulative exposure was 56.3 PCByear (IQR 16.4–237). Cox regression models adjusted for age, ethnicity, parity, and calendar time revealed no association between increasing airborne PCB exposure and risk of any gynecological outcome: UL (HR = 0.99; 95% CI 0.96–1.02), endometriosis (HR = 0.96; 95% CI 0.90–1.02), and PCOS (HR = 0.98; 95% CI 0.93–1.03). Sensitivity analyses addressing potential confounding, misclassification, lag times, and changes in follow-up did not materially alter results.^(49)^

Women exposed to PFAS-contaminated drinking water in Ronneby, Sweden, showed a higher incidence of reproductive disorders compared to non-exposed residents. Among women aged 20–50 years, high PFAS exposure, dominated by PFOS and PFHxS, was significantly associated with increased risk of PCOS (HR = 2.18; 95% CI: 1.43–3.34), while a smaller, non-significant elevation was observed for uterine leiomyoma (HR = 1.28; 95% CI: 0.95–1.74). No increased risk was detected for endometriosis (HR = 0.74; 95% CI: 0.42–1.29). Overall, 27% of women in the cohort had ever lived in areas receiving highly contaminated water, and these individuals accounted for 56 PCOS cases, 332 leiomyoma cases, and 105 endometriosis cases. These findings support a link between high PFAS exposure and PCOS risk, with limited evidence for leiomyoma and none for endometriosis.^(49)^

Maternal exposure to per- and polyfluoroalkyl substances (PFAS) during early pregnancy has also been investigated as a potential developmental contributor to PCOS. In the Project Viva cohort, Wang et al.^(43)^ evaluated six PFAS in maternal plasma and assessed PCOS-related outcomes in adolescent daughters. The study reported that higher prenatal concentrations of 2-(N-ethyl-perfluorooctane sulfonamido) acetate (EtFOSAA) were associated with more than a twofold increase in the odds of self-reported PCOS in offspring, while maternal PFNA levels were linked to moderate-to-severe acne, a marker of hyperandrogenism. No significant associations were observed for menstrual irregularity or hirsutism, and PFAS mixture models did not reveal overall effects. These findings suggest that specific PFAS congeners, rather than cumulative PFAS exposure, may influence the developmental programming of PCOS-related traits.^(44)^

A comparative analysis from Gujarat, India, further highlights how environmental context modifies endocrine disruption in PCOS. Patel et al.^(45)^ evaluated serum concentrations of BPA, MEHP, and DEHP in 40 women with PCOS recruited from urban and rural settings. Urban participants exhibited markedly higher BPA and DEHP levels, reflecting greater industrial and lifestyle-related exposure. In contrast, rural women displayed stronger hormonal perturbations associated with phthalates: DEHP showed robust positive associations with estradiol and inverse associations with prolactin and DHEAS, while MEHP was negatively correlated with DHEAS. These findings underscore that not only the magnitude but the biological consequences of EDC exposure differ by environmental setting, suggesting that geographical context may shape endocrine vulnerability in PCOS.^(45)^

## Discussion

The findings of this systematic review highlight the significant role of endocrine disruptors (EDCs) in the pathophysiology of polycystic ovary syndrome (PCOS). Bisphenol A (BPA) emerged as the most frequently studied EDC, with consistent evidence linking it to hormonal dysregulation, insulin resistance, and hyperandrogenism in women with PCOS. These effects may be attributed to BP's ability to mimic estrogen and interfere with steroidogenesis.^(70)^ Similarly, phthalates, triclosan, and cadmium were shown to disrupt hormonal balance, contributing to metabolic disturbances and ovarian dysfunction.

Currently, there are systematic reviews that relate EDC and PCOS, such as Srnovršnik et al.,^(71)^ which sought the association of EDCs (bisphenols, parabens, and triclosan) with PCOS from studies published between 2007 and 2022. Among its results, it stands out that the highest percentage of studies sought the association of BPA with polycystic ovary syndrome, finding a strong link associated with the presence of negative effects on human ovaries. However, it was also observed that parabens and triclosan were only studied in a single article each, without observing significant associations with PCOS.^(72)^ These results are consistent with those reported by our study, with BPA being the most reported study currently in the literature, and it is related to morphological changes in the ovary as well as evident metabolic alterations. On the other hand, parabens were not included in this review, and triclosan was not conclusive with the presence of PCOS.

Hu et al.,^(73)^ described in their review and meta-analysis that patients with PCOS had significantly higher BPA levels than control groups, and it was also involved in metabolic alterations such as insulin resistance and hyperandrogenism in PCOS. Conclusions like those reported in this work.^(73)^ Phthalate and its association with PCOS were also studied by Neuvonen et al.,^(74)^ in a systematic review; their results did not find an association between PCOS and phthalate exposure. Thus, the results are inconclusive; however, this does not rule out the possible effects of this EDC on the etiopathogenesis of this disease.^(74)^ On the other hand, our work did show an association and a role in the development of PCOS, but the process by which it participates in hormonal dysregulation and the presence of clinical manifestations is still being studied.

On the other hand, Daza-Rodriguez et al.,^(75)^ in a systematic review, described the relationship between infertility and triclosan. These authors find that TC could be implicated in some cases of PCOS, showing a positive relationship with infertile women. However, the results are not conclusive.^(75)^ These findings are like ours, because the information is scarce and does not allow a comparison between articles.

This review presents some limitations, one being the inability to fully cover all currently known EDCs and their relationship with PCOS, due to the limited scientific evidence available and the current lack of interest in the topic. Moreover, there is heterogeneity among the included articles, varying in their designs, study populations, interventions or outcome measures, objectives, and results, making it difficult to compare the different findings.

This review underscores the significant practical implications of endocrine disruptor exposure for PCOS, as evidenced by numerous epidemiological studies. PCOS, being a critical social, mental, and physical health issue among women worldwide, necessitates that physicians recognize environmental pollutants like endocrine disruptors as potential contributors to this condition. The reviewed studies highlight the hormonal imbalance of endocrine disruptors, providing valuable information for the field of toxicology. Additionally, these findings stress the importance of acknowledging the adverse effects of common chemicals on human health, supporting the need for continued research and regulatory actions to protect public well-being.

Our results suggest that direct exposure to a wide range of endocrine disruptors can result in endocrinopathies in healthy women, thereby increasing the current risk of developing these diseases due to their continued use in the general population, particularly in PCOS. This underscores the need to identify more endocrine disruptors and their relationship with PCOS, which could guide the implementation of public health measures to reduce and prevent their presence in products. Although many aspects of this disease still need to be studied, the adoption of preventive measures can help mitigate the burden of morbidity associated with this condition.

## Conclusion

The impact of endocrine disruptors (EDCs) on the development of PCOS has become increasingly recognized, with new substances continuously being linked to the condition. Despite significant progress, research remains limited. BPA stands out as the most closely related EDCs to PCOS, but other disruptors like phthalate, triclosan, cadmium, perfluorooctane sulfonate, perfluorooctanoate, octocrylene, and polychlorinated biphenyls also warrant close attention. Future efforts should emphasize identifying new eDs linked to PCOS to alleviate the public health burden this condition poses for young women. Such efforts should include addressing the complications and comorbidities associated with PCOS while adopting a holistic approach to managing both metabolic and psychological symptoms. This will ensure more effective treatment and improved outcomes.

## Data Availability

The research data are described in the article presented.

## References

[1] Costa CS, Oliveira TF, Freitas-Lima LC, Padilha AS, Krause M, Carneiro MT (2021). Subacute cadmium exposure disrupts the hypothalamic-pituitary-gonadal axis, leading to polycystic ovarian syndrome and premature ovarian failure features in female rats. Environ Pollut.

[2] Luo Y, Nie Y, Tang L, Xu CC, Xu L (2020). The correlation between UDP-glucuronosyltransferase polymorphisms and environmental endocrine disruptors levels in polycystic ovary syndrome patients. Medicine (Baltimore).

[3] Palomba S, Santagni S, Falbo A, La Sala GB (2015). Complications and challenges associated with polycystic ovary syndrome: current perspectives. Int J Womens Health.

[4] Deswal R, Narwal V, Dang A, Pundir C (2020). The prevalence of polycystic ovary syndrome: a brief systematic review. J Hum Reprod Sci.

[5] Chang S, Dunaif A (2021). Diagnosis of polycystic ovary syndrome. Endocrinol Metab Clin North Am.

[6] Witchel SF, Oberfield SE, Peña AS (2019). Polycystic ovary syndrome: pathophysiology, presentation, and treatment with emphasis on adolescent girls. J Endocr Soc.

[7] Louwers YV, Laven JS (2020). Characteristics of polycystic ovary syndrome throughout life. Ther Adv Reprod Health.

[8] Rothenberg SS, Beverley R, Barnard E, Baradaran-Shoraka M, Sanfilippo JS (2018). Polycystic ovary syndrome in adolescents. Best Pract Res Clin Obstet Gynaecol.

[9] Palioura E, Kandaraki E, Diamanti-Kandarakis E (2014). Endocrine disruptors and polycystic ovary syndrome: a focus on Bisphenol A and its potential pathophysiological aspects. Horm Mol Biol Clin Investig.

[10] Kabir ER, Rahman MS, Rahman I (2015). A review on endocrine disruptors and their possible impacts on human health. Environ Toxicol Pharmacol.

[11] Monneret C (2017). What is an endocrine disruptor?. C R Biol.

[12] Encarnação T, Pais AA, Campos MG, Burrows HD (2019). Endocrine disrupting chemicals: impact on human health, wildlife, and the environment. Sci Prog.

[13] Annamalai J, Namasivayam V (2015). Endocrine disrupting chemicals in the atmosphere: their effects on humans and wildlife. Environ Int.

[14] Yilmaz B, Terekeci H, Sandal S, Kelestimur F (2020). Endocrine disrupting chemicals: exposure, effects on human health, mechanism of action, models for testing and strategies for prevention. Rev Endocr Metab Disord.

[15] Rattan S, Zhou C, Chiang C, Mahalingam S, Brehm E, Flaws JA (2017). Exposure to endocrine disruptors during adulthood: consequences for female fertility. J Endocrinol.

[16] Di Pietro G, Forcucci F, Chiarelli F (2023). Endocrine disruptor chemicals and children's health. Int J Mol Sci.

[17] Papalou O, Kandaraki EA, Papadakis G, Diamanti-Kandarakis E (2019). Endocrine disrupting chemicals: an occult mediator of metabolic disease. Front Endocrinol (Lausanne).

[18] Marconetto A, Babini A, Ñañez M, Moreno L, Rosato O, Fux Otta C (2022). Principales disruptores endocrinos vinculados con salud reproductiva femenina: bases biológicas de su asociación. Medicina (B Aires).

[19] Page MJ, McKenzie JE, Bossuyt PM, Boutron I, Hoffmann TC, Mulrow CD (2021). The PRISMA 2020 statement: an updated guideline for reporting systematic reviews. BMJ.

[20] Aromataris E, Lockwood C, Porritt K, Pilla B, Jordan Z (2024). JBI Manual for Evidence Synthesis.

[21] Ouzzani M, Hammady H, Fedorowicz Z, Elmagarmid A (2016). Rayyan-a web and mobile app for systematic reviews. Syst Rev.

[22] Tarantino G, Valentino R, Di Somma C, D’Esposito V, Passaretti F, Pizza G (2013). Bisphenol A in polycystic ovary syndrome and its association with liver–spleen axis. Clin Endocrinol (Oxf).

[23] Vagi SJ, Azziz-Baumgartner E, Sjödin A, Calafat AM, Dumesic D, Gonzalez L (2014). Exploring the potential association between brominated diphenyl ethers, polychlorinated biphenyls, organochlorine pesticides, perfluorinated compounds, phthalates, and bisphenol a in polycystic ovary syndrome: a case-control study. BMC Endocr Disord.

[24] Akın L, Kendirci M, Narin F, Kurtoglu S, Saraymen R, Kondolot M (2015). The endocrine disruptor bisphenol A may play a role in the aetiopathogenesis of polycystic ovary syndrome in adolescent girls. Acta Paediatr.

[25] Vahedi M, Saeedi A, Poorbaghi SL, Sepehrimanesh M, Fattahi M (2016). Metabolic and endocrine effects of bisphenol A exposure in market seller women with polycystic ovary syndrome. Environ Sci Pollut Res.

[26] Zhou W, Fang F, Zhu W, Chen ZJ, Du Y, Zhang J (2016). Bisphenol A and ovarian reserve among infertile women with polycystic ovarian syndrome. Int J Environ Res Public Health.

[27] Hossein Rashidi B, Amanlou M, Behrouzi Lak T, Ghazizadeh M, Haghollahi F, Bagheri M (2017). The association between Bisphenol A and polycystic ovarian syndrome: a case-control study. Acta Med Iran.

[28] Jin Y, Zhang Q, Pan JX, Wang FF, Qu F (2019). The effects of di(2-ethylhexyl) phthalate exposure in women with polycystic ovary syndrome undergoing in vitro fertilization. J Int Med Res.

[29] Ye J, Zhu W, Liu H, Mao Y, Jin F, Zhang J (2018). Environmental exposure to triclosan and polycystic ovary syndrome: a cross-sectional study in China. BMJ Open.

[30] Konieczna A, Rachoń D, Owczarek K, Kubica P, Kowalewska A, Kudłak B (2018). Serum bisphenol A concentrations correlate with serum testosterone levels in women with polycystic ovary syndrome. Reprod Toxicol.

[31] Gu J, Yuan T, Ni N, Ma Y, Shen Z, Yu X (2019). Urinary concentration of personal care products and polycystic ovary syndrome: a case-control study. Environ Res.

[32] Akgül S, Sur Ü, Düzçeker Y, Balcı A, Kızılkan MP, Kanbur N (2019). Bisphenol A and phthalate levels in adolescents with polycystic ovary syndrome. Gynecol Endocrinol.

[33] Akın L, Kendirci M, Narin F, Kurtoglu S, Hatipoğlu N, Elmalı F (2020). Endocrine disruptors and polycystic ovary syndrome: phthalates. J Clin Res Pediatr Endocrinol.

[34] Šimková M, Vítků J, Kolátorová L, Vrbíková J, Vosátková M, Včelák J (2020). Endocrine disruptors, obesity, and cytokines - how relevant are they to PCOS?. Physiol Res.

[35] Kim K, Pollack AZ, Nobles CJ, Sjaarda LA, Zolton JR, Radoc JG (2021). Associations between blood cadmium and endocrine features related to PCOS-phenotypes in healthy women of reproductive age: a prospective cohort study. Environ Health.

[36] Lazúrová Z, Figurová J, Hubková B, Mašlanková J, Lazúrová I (2021). Urinary bisphenol A in women with polycystic ovary syndrome – a possible suppressive effect on steroidogenesis?. Horm Mol Biol Clin Investig.

[37] Al-Saleh I (2022). The relationship between urinary phthalate metabolites and polycystic ovary syndrome in women undergoing in vitro fertilization: nested case-control study. Chemosphere.

[38] Zhang M, Liu C, Yuan XQ, Cui FP, Miao Y, Yao W (2023). Individual and joint associations of urinary phthalate metabolites with polycystic ovary and polycystic ovary syndrome: results from the TREE cohort. Environ Toxicol Pharmacol.

[39] Majewska J, Berg A, Jurewicz J, Owczarek K, Zajdel R, Kilanowicz A (2024). Bisphenol A analogues and metabolic syndrome in women with polycystic ovary syndrome. Reprod Toxicol.

[40] Zhan W, Qiu W, Ao Y, Zhou W, Sun Y, Zhao H (2023). Environmental exposure to emerging alternatives of per- and polyfluoroalkyl substances and polycystic ovarian syndrome in women diagnosed with infertility: a mixture analysis. Environ Health Perspect.

[41] Guo Z, Qiu H, Wang L, Wang L, Wang C, Chen M (2017). Association of serum organochlorine pesticides concentrations with reproductive hormone levels and polycystic ovary syndrome in a Chinese population. Chemosphere.

[42] Liang C, Zhang Z, Cao Y, Wang J, Shen L, Jiang T (2022). Exposure to multiple toxic metals and polycystic ovary syndrome risk: endocrine disrupting effect from As, Pb and Ba. Sci Total Environ.

[43] Wang Z, Fleisch A, Rifas-Shiman SL, Calafat AM, James-Todd T, Coull BA (2025). Associations of maternal per- and polyfluoroalkyl substance plasma concentrations during pregnancy with offspring polycystic ovary syndrome and related characteristics in project viva. Environ Res.

[44] Kawa IA, Masood A, Ganie MA, Fatima Q, Jeelani H, Manzoor S (2019). Bisphenol A (BPA) acts as an endocrine disruptor in women with Polycystic Ovary Syndrome: hormonal and metabolic evaluation. Obes Med.

[45] Patel J, Chaudhary H, Panchal S, Joshi T, Joshi R (2024). Endocrine-disrupting chemicals and hormonal profiles in PCOS women: a comparative study between urban and rural environment. Reprod Toxicol.

[46] Milanović M, Milošević N, Sudji J, Stojanoski S, Atanacković Krstonošić M, Bjelica A (2020). Can environmental pollutant bisphenol A increase metabolic risk in polycystic ovary syndrome?. Clin Chim Acta.

[47] Milankov A, Milanović M, Milošević N, Sudji J, Pejaković S, Milić N (2023). The effects of phthalate exposure on metabolic parameters in polycystic ovary syndrome. Clin Chim Acta.

[48] Jurewicz J, Majewska J, Berg A, Owczarek K, Zajdel R, Kaleta D (2021). Serum bisphenol A analogues in women diagnosed with the polycystic ovary syndrome – is there an association?. Environ Pollut.

[49] Tøttenborg SS, Wise LA, Wesselink AK, Nielsen HS, Petersen KU, Fox MP (2025). Exposure to airborne polychlorinated biphenyls and risk of uterine leiomyomata, endometriosis, and polycystic ovarian syndrome: a register-based Danish cohort study. Environ Toxicol Pharmacol.

[50] Hammarstrand S, Jakobsson K, Andersson E, Xu Y, Li Y, Olovsson M (2021). Perfluoroalkyl substances (PFAS) in drinking water and risk for polycystic ovarian syndrome, uterine leiomyoma, and endometriosis: a Swedish cohort study. Environ Int.

[51] Brennan E, Butler AE, Drage DS, Sathyapalan T, Atkin SL (2023). Serum polychlorinated biphenyl levels and circulating miRNAs in non-obese women with and without polycystic ovary syndrome. Front Endocrinol (Lausanne).

[52] Prabhu NB, Vasishta S, Bhat SK, Joshi MB, Kabekkodu SP, Satyamoorthy K (2023). Distinct metabolic signatures in blood plasma of bisphenol A–exposed women with polycystic ovarian syndrome. Environ Sci Pollut Res.

[53] Abraham A, Chakraborty P (2019). A review on sources and health impacts of bisphenol A. Rev Environ Health.

[54] Gramec Skledar D, Peterlin Mašič L (2016). Bisphenol A and its analogs: do their metabolites have endocrine activity?. Environ Toxicol Pharmacol.

[55] Kechagias KS, Semertzidou A, Athanasiou A, Paraskevaidi M, Kyrgiou M (2020). Bisphenol-A and polycystic ovary syndrome: a review of the literature. Rev Environ Health.

[56] Calvo Gutierrez H (2023). Revisión sistemática: el tóxico silencioso de la vida moderna bisfenol A. Rev Invest Inform Salud.

[57] Stavridis K, Triantafyllidou O, Pisimisi M, Vlahos N (2022). Bisphenol-A and female fertility: an update of existing epidemiological studies. J Clin Med.

[58] Hlisníková H, Petrovičová I, Kolena B, Šidlovská M, Sirotkin A (2020). Effects and mechanisms of phthalates’ action on reproductive processes and reproductive health: a literature review. Int J Environ Res Public Health.

[59] Xu C, Lin H, Zhao Y, Zhang Y (2011). Determination of serum levels of three phthalate esters in patients with polycystic ovary syndrome. Sci Res Essays.

[60] Pan J, Liu P, Yu X, Zhang Z, Liu J (2024). The adverse role of endocrine disrupting chemicals in the reproductive system. Front Endocrinol (Lausanne).

[61] Özel Ş, Tokmak A, Aykut O, Aktulay A, Hançerlioğulları N, Engin Ustun Y (2019). Serum levels of phthalates and bisphenol-A in patients with primary ovarian insufficiency. Gynecol Endocrinol.

[62] Rozati R, Fatima S (2019). Etiological role of environmental toxicants in polycystic ovarian syndrome. Int J Res Rev.

[63] Zheng G, Wang L, Guo Z, Sun L, Wang L, Wang C (2015). Association of serum heavy metals and trace element concentrations with reproductive hormone levels and polycystic ovary syndrome in a Chinese population. Biol Trace Elem Res.

[64] Chedrese PJ, Piasek M, Henson MC (2006). Cadmium as an endocrine disruptor in the reproductive system. Immunol Endocr Metab Agents Med Chem.

[65] Maksymowicz M, Ręka G, Machowiec P, Piecewicz-Szczęsna H (2022). Impact of triclosan on female and male reproductive system and its consequences on fertility: a literature review. J Family Reprod Health.

[66] Jurewicz J, Wielgomas B, Radwan M, Karwacka A, Klimowska A, Dziewirska E (2019). Triclosan exposure and ovarian reserve. Reprod Toxicol.

[67] Zhang QY, Ma XY, Wang XC, Ngo HH (2016). Assessment of multiple hormone activities of a UV-filter (octocrylene) in zebrafish (Danio rerio). Chemosphere.

[68] Ding N, Harlow SD, Randolph JF, Loch-Caruso R, Park SK (2020). Perfluoroalkyl and polyfluoroalkyl substances (PFAS) and their effects on the ovary. Hum Reprod Update.

[69] Rickard BP, Rizvi I, Fenton SE (2022). Per- and poly-fluoroalkyl substances (PFAS) and female reproductive outcomes: PFAS elimination, endocrine-mediated effects, and disease. Toxicology.

[70] Mokra K (2021). Endocrine disruptor potential of short- and long-chain perfluoroalkyl substances (PFASs)-a synthesis of current knowledge with proposal of molecular mechanism. Int J Mol Sci.

[71] Srnovršnik T, Virant-Klun I, Pinter B (2023). Polycystic ovary syndrome and endocrine disruptors (Bisphenols, Parabens, and Triclosan)-a systematic review. Life.

[72] Gregoraszczuk EL, Ptak A (2013). Endocrine-disrupting chemicals: some actions of POPs on female reproduction. Int J Endocrinol.

[73] Hu Y, Wen S, Yuan D, Peng L, Zeng R, Yang Z (2018). The association between the environmental endocrine disruptor bisphenol A and polycystic ovary syndrome: a systematic review and meta-analysis. Gynecol Endocrinol.

[74] Neuvonen R, Huovinen M, Dorman DC, Laitinen H, Sahlman H (2023). Phthalates and polycystic ovary syndrome – Systematic literature review. Reprod Toxicol.

[75] Daza-Rodríguez B, Aparicio-Marenco D, Márquez-Lázaro J (2023). Association of triclosan and human infertility: a systematic review. Environ Anal Health Toxicol.

