# Supplementary material for: Association between exposure to endocrine-disrupting chemicals and polycystic ovary syndrome: a systematic review
Source: Rev Bras Ginecol Obstet. 2026 May 29;48:e-rbgo19. doi: 10.61622/rbgo/2026rbgo19 (PMC13399378; doi:10.61622/rbgo/2026rbgo19)
Supplement: Supplementary Material [file 1806-9339-rbgo-48-e-rbgo19-Suppl01.pdf]

## Supplementary Material

**Table 1S.** Search strategy

| **PubMed** |
|---|
| ("Polycystic Ovary Syndrome"[Mesh] OR (Ovary Syndrome, Polycystic ) OR (Syndrome, Polycystic Ovary) OR (Stein-Leventhal Syndrome) OR (Stein Leventhal Syndrome) OR (Syndrome, Stein-Leventhal) OR (Sclerocystic Ovarian Degeneration) OR (Ovarian Degeneration, Sclerocystic) OR (Sclerocystic Ovary Syndrome) OR (polycystic ovarian syndrome) OR (Ovarian Syndrome, Polycystic) OR (Polycystic Ovary Syndrome 1) OR (Sclerocystic Ovaries) OR (Ovary, Sclerocystic) OR (Sclerocystic Ovary) OR (PCOS) AND (2013:2025[pdat])) AND ("Endocrine Disruptors"[Mesh] OR (Disruptors, Endocrine) OR (Endocrine Disrupting Chemicals) OR (Chemicals, Endocrine Disrupting) OR (Endocrine Disruptor) OR (Disruptor, Endocrine) OR (Endocrine Disrupting Chemical) OR (Chemical, Endocrine Disrupting) OR (Disrupting Chemical, Endocrine) OR (Endocrine Disruptor Effect) OR (Disruptor Effect, Endocrine) OR (Effect, Endocrine Disruptor) OR (Endocrine Disruptor Effects) OR (Disruptor Effects, Endocrine) OR (Effects, Endocrine Disruptor) OR ("Triclosan"[Mesh]) OR (Bisphenol A) OR (ECDs) AND (2013:2025[pdat])). From 2013/1/1 to 2025/11/25 |
| **Scopus** |
| TITLE-ABS-KEY ("polycystic ovary syndrome" OR "Polycystic Ovary" OR "PCOS") AND TITLE-ABS-KEY ("Endocrine Disruptor" OR "Bisphenol A" OR "Triclosan" OR "phthalates" OR "cadmium" OR "PFAS") AND PUBYEAR > 2013 AND PUBYEAR < 2026 AND PUBYEAR > 2012 AND PUBYEAR < 2025 |
| **LILACS (DeCS)** |
| (("Síndrome de Ovario Poliquístico" OR "Ovario Poliquístico" OR PCOS) AND ("Disruptores Endocrinos" OR "Bisfenol A" OR triclosán OR ftalatos OR cadmio OR PFAS)) |
| **ScienceDirect** |
| ("polycystic ovary syndrome" OR PCOS) AND ("endocrine disruptor" OR "endocrine disrupting chemicals" OR "bisphenol A" OR triclosan OR phthalate OR cadmium OR PFAS) |
